# Foreign Bodies**: **Aspirated or Ingested? A Report of Two Unusual Cases

**Published:** 2012

**Authors:** Aliasghar Arabi Mianroodi, Yeganeh Teimouri, Neil A. Vallance

**Affiliations:** 1 *Department of Otolaryngology, Head and Neck Surgery, Shafa Hospital, Kerman, Iran*; 2 *Department of Otolaryngology, Head and Neck Surgery, Monash Medical Center, Melbourne****,**** Australia*

**Keywords:** Aspiration, Bronchus, Esophagus, Foreign body

## Abstract

**Introduction:**

The diagnosis of foreign bodies in the upper aerodigestive tract is usually straightforward but sometimes it can be delayed or the location of esophageal and upper airway foreign bodies can be mistakenly interchanged.

**Case Report:**

We present two interesting cases that caused diagnostic challenges which could have led to serious complications if a greater delay in diagnosis had occurred.

**Conclusion:**

In order to diagnose upper aerodigestive tract foreign bodies without delay, a careful history and physical examination with proper X-rays are helpful.

## Introduction

Foreign bodies in the upper aerodigestive tract can be a therapeutic and diagnostic challenge and can be fatal if diagnosis is delayed because of early or late complications. Misdiagnosis due to mistakenly interchanging the location of bronchial and esophageal foreign bodies can happen for longstanding esophageal foreign bodies that have respiratory signs and symptoms. We present two prototypic cases of bronchial and esophageal foreign bodies which were initially misdiagnosed. We will discuss how these kinds of pitfalls can be prevented.

## Case Reports

***Case One***

**Fig 1:**
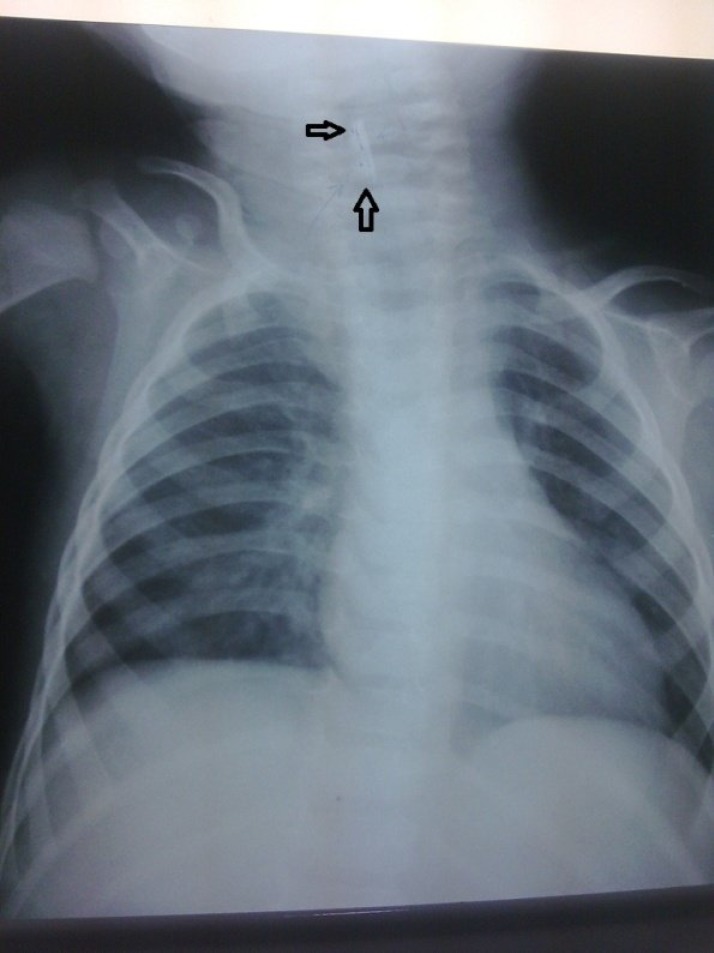
Esophageal foreign body which was misdiagnosed as a tracheal foreign body because of respiratory problems.

A 10-month-old boy was referred to the Ear, Nose and Throat Department at Shafa Hospital, Kerman, Iran, with a complaint of a protracted cough that had lasted for one and a half months. The attending otolaryngologist referred the patient for bronchoscopy and foreign body removal as his diagnosis was the presence of a longstanding tracheal foreign body. The story began after a choking accident which lasted for 1 to 2 minutes. The boy’s mother mentioned that the patient had difficulty swallowing and experienced postprandial vomiting for a few days following the incident. He also suffered from a protracted cough and respiratory infection that did not respond to medical management. During this time the patient had obvious weight loss. His mother did not mention sialorrhea and it was not obvious in his examination. In the primary examination of the patient fine rales could be heard during lung auscultation. Vital signs were stable and all other examination results were normal. An anteroposterior (AP) chest X-ray showed a longitudinal density in front of the C3-C4 region of the spine, which, according to the radiologist’s report, was in favor of a tracheal foreign body (Fig 1).

**Fig 2:**
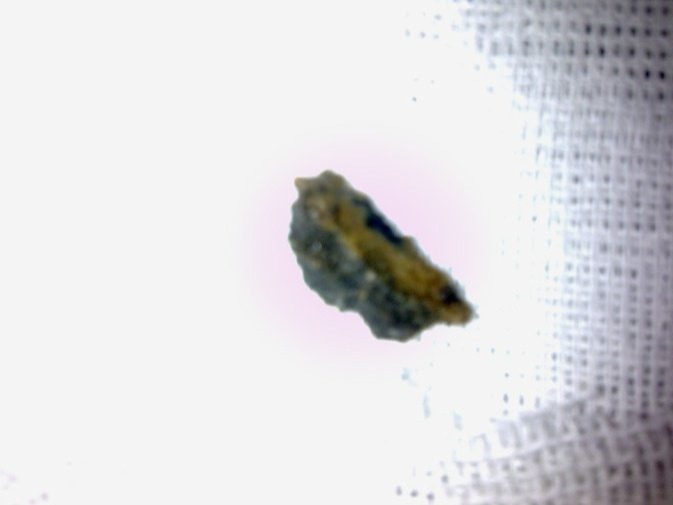
The foreign body removed from the esophagus was a piece of stone.

According to the patient’s history and physical examination he was prepared for bronchoscopy and foreign body removal. Due to the possibility of the presence of an upper esophageal foreign body, an esophagoscopy set up was also available. The bronchoscopy showed nothing in the trachea so an esophagoscopy was performed that showed a 3 cm long stone in the proximal esophagus (Figure 2). The stone was removed uneventfully and the patient discharged the day after the procedure with no problems. He was in good condition in a follow-up examination. 

***Case Two***

A 15-year-old boy was admitted to the Emergency Department at Monash Medical Center, Melbourne, Australia, claiming he had swallowed a homemade dart consisting of a sewing needle in a piece of shoe lace. He was complaining of a scratchy throat after choking for a few seconds. He could talk and swallow well. The patient was examined thoroughly by medical officers who were convinced that the foreign body had passed through the esophagus as a chest X-ray was also normal. He was discharged and asked to come back to have abdominal and chest X-rays if the foreign body had not passed after three days.

**Fig 3:**
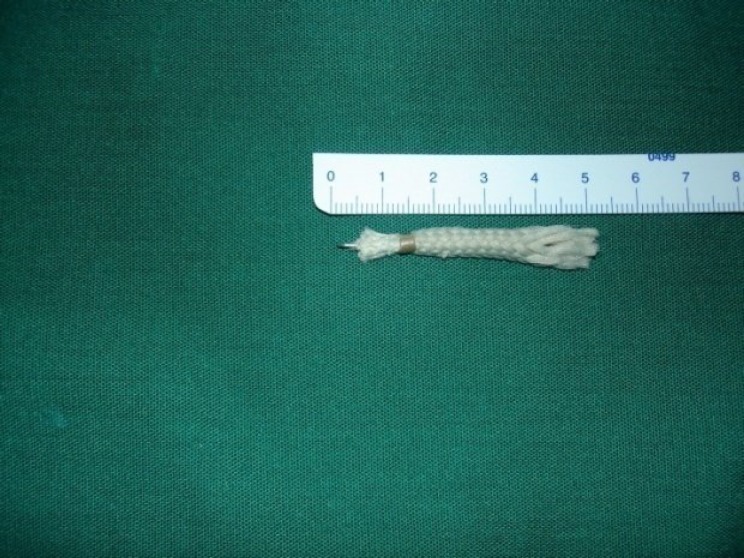
Bronchial foreign body (a needle in a shoelace) that was missed because of lack of respiratory symptoms and the type of foreign body.

The patient came back to the hospital after 24 hours due to a cough that was bothering him. His chest X-ray was reviewed again and this time a density confirming the presence of a needle could be seen in the left main bronchus with no other abnormalities (Fig 3). 

**Fig 4:**
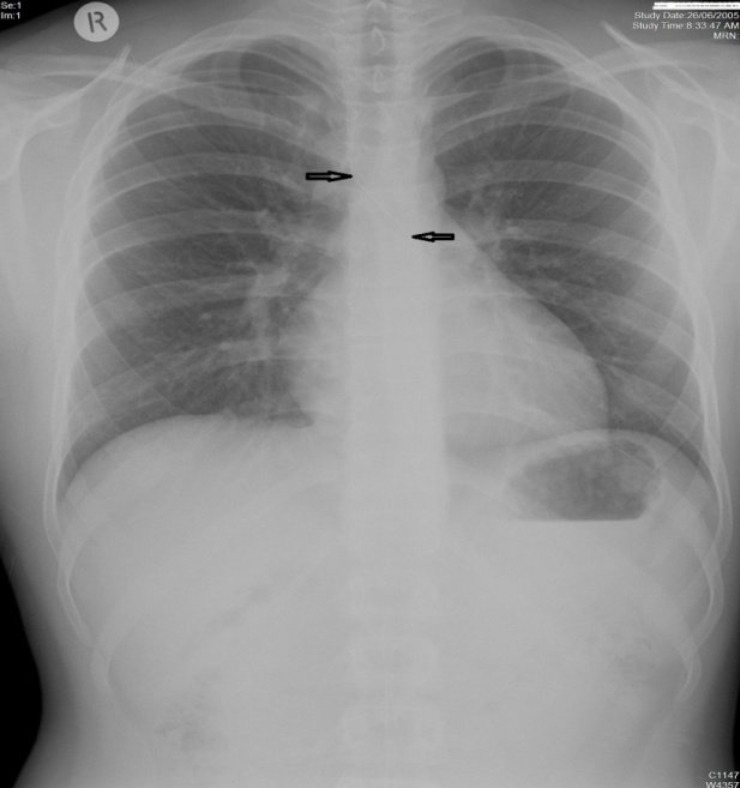
Foreign body removed from the bronchus consisted of a homemade dart.

The patient was admitted to the Otolaryngology Department and was prepared for rigid bronchoscopy and foreign body removal. The foreign body, which was a 5 cm long needle inserted into a piece of shoe lace (Fig 4), was removed from the left main bronchus uneventfully by the main author and the patient was discharged the day after with no problems. 

## Discussion

Foreign bodies in the upper aerodigestive tract cause thousands of cases of morbidity and mortality worldwide each year. In 2003, foreign body aspiration was responsible for 53 deaths according to reports from the Ministry of Health in Iran (1). In the United States this figure was 600 deaths in children in 1987, according to a report by Mannings and colleagues (2), which has decreased to 150 deaths annually in recent years (3). Although the number of deaths per year has decreased they still occur despite increased public awareness of the danger of foreign body aspiration or ingestion and advances in diagnostic and treatment facilities. However, as most of the victims are young children who are playful and may not be witnessed during the aspiration the diagnosis may not be done on time and complications can arise (4,5). The reason for the increased incidence in children is incomplete dentition, limited oromotor control and immature judgment, and also a tendency to explore the surrounding world with their hands and mouths (5). 

The incidence of complications increases as more time passes between inhalation and removal of the foreign body (6). Ingestion of foreign bodies may have unusual symptoms like unexplained fever, sore throat, cough, stridor, wheezing, anorexia, nausea, vomiting, dysphagia, and spatial chest or abdominal pain if they remain for more than seven days (5-7). The patient may even be asymptomatic in up to 35% of cases of gastrointestinal foreign bodies (7). 

The first case reported here had acute-stage symptoms of foreign body aspiration maybe because the foreign body was lodged in the laryngeal inlet and after coughing, it passed to the esophagus where it remained and caused difficulty in swallowing. Foreign bodies in the esophagus do not cause respiratory distress unless they are large and compress the posterior wall of the trachea or, as mentioned earlier, the respiratory symptoms are caused by longstanding lodgment (5-7). 

Differentiating these two entities is possible by obtaining AP and lateral chest X-rays in cases of opaque foreign bodies (3,5,7,8).

In case of radiolucent foreign bodies, X-ray findings are not straightforward and the presence of a esophageal foreign body can be indicated by distortion of the normal anatomy or other complications (8,7). In addition, endoscopy can be used for both diagnostic and therapeutic purposes. The diagnosis of the first case was delayed as it was not witnessed and then it presented with more respiratory symptoms.

The second case is interesting as it shows how easily a large object can be aspirated and can remain asymptomatic. The diagnosis of foreign body aspiration is usually possible when an appropriate history is taken (3). A typical case of foreign body aspiration is a young child who has a choking accident while playing. The acute stage is defined by a cough and respiratory distress. After the foreign body becomes lodged and any reflexes have ceased to occur the patient may be asymptomatic (second phase) until complications happen (third phase) (3). This was what happened in the second case but a troublesome cough in the third phase prompted the patient to come to the emergency department quickly and no complications arose.

Missing a foreign body aspiration is not unusual as the first stage may not be witnessed by parents and the second stage may also be asymptomatic. The correct diagnosis will only be suspected when a careful history is taken that highlights a protracted cough and the occurrence of a choking incident.

## Conclusion

A careful history, thorough physical exam, and two proper X-rays, especially two different X-ray views (AP and lateral), can help to diagnose foreign bodies in the upper airway early and prevent complication.
